# Coupled reactions by coupled enzymes: alcohol to lactone cascade with alcohol dehydrogenase–cyclohexanone monooxygenase fusions

**DOI:** 10.1007/s00253-017-8501-4

**Published:** 2017-09-15

**Authors:** Friso S. Aalbers, Marco W. Fraaije

**Affiliations:** Molecular Enzymology Group, Groningen Biomolecular Sciences and Biotechnology Institute, University of Groningen, Nijenborgh 4, 9747 AG Groningen, The Netherlands

**Keywords:** Enzyme fusion, Cascade, Cyclohexanone monooxygenase, Alcohol dehydrogenase

## Abstract

The combination of redox enzymes for redox-neutral cascade reactions has received increasing appreciation. An example is the combination of an alcohol dehydrogenase (ADH) with a cyclohexanone monooxygenase (CHMO). The ADH can use NADP^+^ to oxidize cyclohexanol to form cyclohexanone and NADPH. Both products are then used by CHMO to produce ε-caprolactone. In this study, these two redox-complementary enzymes were fused, to create a self-sufficient bifunctional enzyme that can convert alcohols to esters or lactones. Three different ADH genes were fused to a gene coding for a thermostable CHMO, in both orientations (ADH-CHMO and CHMO-ADH). All six fusion enzymes could be produced and purified. For two of the three ADHs, we found a clear difference between the two orientations: one that showed the expected ADH activity, and one that showed low to no activity. The ADH activity of each fusion enzyme correlated with its oligomerization state. All fusions retained CHMO activity, and stability was hardly affected. The TbADH-TmCHMO fusion was selected to perform a cascade reaction, producing ε-caprolactone from cyclohexanol. By circumventing substrate and product inhibition, a > 99% conversion of 200 mM cyclohexanol could be achieved in 24 h, with > 13,000 turnovers per fusion enzyme molecule.

**Electronic supplementary material:**

The online version of this article (10.1007/s00253-017-8501-4) contains supplementary material, which is available to authorized users.

## Introduction

The use of enzymes for industrial-scale production of chemicals has great potential. Enzymatic reactions allow the production of specific compounds without the use of harsh chemicals, organic solvents or extreme conditions, such as high temperature and high pressure. With multi-step enzymatic reactions, also referred to as cascade reactions, complex compounds can be produced in a single setup, with minimal input and without the need of isolating intermediates (Muschiol et al. 2015; Xue and Woodley 2012).

There are several problems preventing the large-scale industrial application of enzymatic reactions (Woodley 2013). One important hurdle is stability; enzymes evolved to catalyze reactions at the level of a living cell, and cannot maintain production for long periods of time, with co-solvents, and at higher temperatures. Another frequently encountered issue is insufficient functional expression, in particular when the enzyme which presents the rate-limiting step in a cascade has a limited level of expression. Furthermore, general availability of enzymes can be an issue, and to improve expression of enzymes individually is comparatively costly.

An approach that could address some of these difficulties is producing catalytically complementary enzymes fused to each other. Fusions are generally created by organizing a single open reading frame coding for two or more enzymes, from which the translation would result in a multifunctional enzyme. Some studies on enzyme fusions have found various advantages, including improved catalytic efficiency, expression, folding and stability (Chen et al. 2013; Yang et al. 2016). In some cases, as the fused enzymes are in close proximity, a product molecule from one enzyme could directly migrate to the active site of the second enzyme. This phenomenon is referred to as substrate channeling (Wheeldon et al. 2016). In this way, a cascade reaction with fused enzymes can be more efficient (Iturrate et al. 2010). Although some studies have found particular benefits from fused enzymes, it is not clear how to design an efficient and stable fusion. The current state of the art of fusions has been reviewed recently (Elleuche 2015; Yang et al. 2016).

**Fig. 1:**
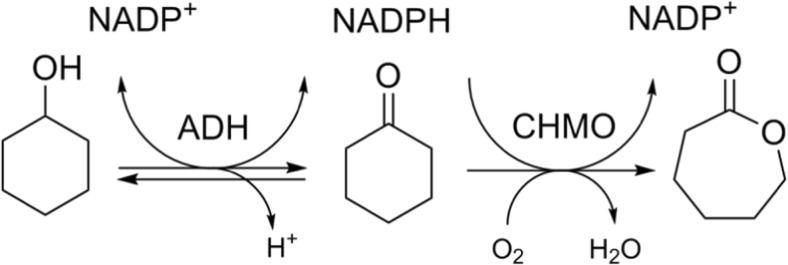
Cascade reaction from cyclohexanol to ɛ-carpolactone, involving an alcohol dehydrogenase (ADH) and a cyclohexanone monooxygenase (CHMO)

The aim of this study is to investigate fusion engineering as an approach to co-express and improve biocatalysts in terms of stability and catalytic efficiency. We fused alcohol dehydrogenases (ADHs, EC 1.1.1) to a Baeyer-Villiger monooxygenase (BVMO, EC 1.14.13), as the two types of enzymes can perform various cascade reactions together. ADHs can oxidize alcohols to ketones, whereby NADPH is formed, and a BVMO can then use the NADPH and oxygen to oxidize the ketones to lactones or esters. The cascade reaction we aimed to optimize with this approach is the biocatalytic production of ε-caprolactone from cyclohexanol (Fig. 1), which has been targeted in various recent studies (Mallin et al. 2013; Staudt et al. 2013; Sattler et al. 2014; Schmidt et al. 2015).

The BVMO that was used for each fusion is the recently characterized cyclohexanone monooxygenase from *Thermocrispum municipale* (TmCHMO) (Romero et al. 2016). The CHMO is more thermostable and has higher solvent tolerance than the commonly used AcCHMO from *Acinetobacter calcoaceticus* NCIMB 9871. Therefore, the TmCHMO is a promising biocatalyst for industrial application. Three ADH enzymes were chosen as fusion partners for the TmCHMO, based on their preference for NADP^+^ and thermostability. Two are known thermostable enzymes: TbADH from *Thermoanaerobacter brockii* (Lamed et al. 1981) and ADHA from *Pyrococcus furiosus* (Van der Oost et al. 2001). The other one is a newly discovered alcohol dehydrogenase: ADHMi from *Mesotoga infera*. We identified the respective gene of the latter ADH by searching the genome sequence database for predicted proteins showing sequence similarity with annotated cyclohexanol dehydrogenases while being from a mesophile, and predicted to accept NADP^+^ as coenzyme using Cofactory (Geertz-Hansen et al. 2014).

Fusions between the ADHs and TmCHMO were made in both orientations, with the TmCHMO either at the C-terminus or at the N-terminus of the ADH (Table 1). In this way, six fusion constructs were created in total. The rationale for this approach is that either of the enzymes could be hindered by its fusion partner in one orientation. In particular, ADHs form oligomers, and the oligomerization may be obstructed, which could decrease their stability (Korkhin et al. 1999), and could prevent their activity (Breiter et al. 1994). The two enzymes are connected by a glycine-rich linker, which in a previous study on fusions was found to be the most favorable among the tested linkers (Jeon et al. 2015).

**Table 1:** Fusion constructs

Enzyme	N-terminal	Linker	C-terminal	Mw (kDa)
A-Tm	ADHA	Glycine-rich	TmCHMO	90
Tm-A	TmCHMO	Glycine-rich	ADHA	90
Mi-Tm	ADHMi	Glycine-rich	TmCHMO	91
Tm-Mi	TmCHMO	Glycine-rich	ADHMi	91
Tb-Tm	TbADH	Glycine-rich	TmCHMO	102
Tm-Tb	TmCHMO	Glycine-rich	TbADH	102

Each fusion construct also contained a His-tag at the N-terminal side. Molecular weight of each fusion is based on predicted size and SDS-PAGE. Glycine-rich linker amino acid sequence: SGGSGGSGGSAG

We intended to create a fusion that can convert cyclohexanol to ε-caprolactone in vitro, which is stable for a long period of time at elevated temperatures, leading to high total turnover numbers. Activity of both enzymes of the fusions was determined, and their stability and oligomerization were studied. The best candidate was used to perform conversions of cyclohexanol into ε-caprolactone.

## Materials and methods

### Materials, strains and culture media

Oligonucleotide primers for cloning and mutagenesis were ordered from Sigma-Aldrich. For amplification of the gene inserts, PfuUltra II HotStart PCR master mix was used, purchased from Agilent Technologies. Other chemicals were purchased from Sigma-Aldrich and Acros Organics. Precast native PAGE gradient gels were ordered from Bio-Rad. CAL-A lipase was ordered from c-LEcta. As host strain for recombinant DNA, *Escherichia coli* NEB® 10-beta (New England Biolabs) was used. Precultures were grown in lysogeny broth (LB), and the subsequent main cultures in terrific broth (TB).

### Cloning of fusion constructs

The *TmCHMO* gene was amplified from an in-house plasmid (pBAD-His-Tag-SUMO-TmCHMO; Romero et al. 2016) using PCR with two primer sets that have different flanking regions which contain restriction sites (Table S1). After PCR purification, the two obtained fragments were separately cloned into a pBAD vector. This vector has a region coding for a glycine-rich linker (SGGSGGSGGSAG), which is between two cloning sites, and the vector has an N-terminal His-tag. One PCR fragment was inserted into the *Nde*I-*Xho*I site of one vector, and the other between the *Pvu*II and *Hin*dIII sites of another vector, using restriction and ligation. In this way, two vectors were obtained: one containing the *TmCHMO* gene at the N-terminal side of the glycine-rich linker, and one at the C-terminal side. To multiply the two vectors, the recombinant plasmids were used to transform chemically competent *E. coli* NEB® 10-beta cells. To identify colonies that had the desired insert, colony PCR was done, using a gene-specific forward and a vector-specific reverse primer. Colonies that gave PCR product were subsequently analyzed using Sanger sequencing (GATC Biotech). Cells harboring the correct plasmids were stored at − 80 °C in 25% glycerol. For subsequent subcloning of the three ADH genes, the two vectors that contain the *TmCHMO* gene were used as backbones. The *Tbadh* (X64841.1) gene was obtained from an in-house plasmid (pBad::HheC::AdhT; Szymanski et al. 2010); a plasmid containing the *adhA* gene was previously received from the laboratory of microbiology at Wageningen University (Van der Oost et al. 2001); *adhMi* (CCU83614.1) was ordered as a codon-optimized synthetic gene (MF276894) and was subcloned from the supplied standard vector (GenScript, USA). The genes were amplified using two primer sets (Table S1), and all the other steps described above were repeated for each ADH gene, with a few exceptions. To avoid digestion in the middle of either gene of one construct, first, *adhA* was cloned into the starting vector at the *Pvu*II-*Apa*I site, and subsequently, the *TmCHMO* gene was inserted at the *Nde*I-*Xho*I site. Thus, six fusion constructs were cloned that have either the ADH gene or TmCHMO gene at the N-terminal side, and the other gene at the C-terminal end. For expression of the individual *adh* genes, fusion constructs with the *adh* upstream of the linker region were mutated with QuickChange primers, thereby introducing a stop codon at the end of each *adh* gene.

### Expression and protein purification

*E. coli* cells harboring the plasmids containing a gene fusion were grown overnight at 37 °C in 5 mL LB and 50 μg/mL ampicillin. The precultures were inoculated from the − 80 °C glycerol stocks. On the next day, the precultures were used to inoculate 50 mL of TB, containing 50 μg/mL ampicillin and 0.02% (*w*/*v*) l-arabinose, in baffled flasks. The 50 mL cultures were incubated at 24 °C for 40 h. The cells were then harvested (3000×*g*, 4 °C), and then resuspended in 10 mL of 50 mM Tris/HCl pH 8.0. Resuspended or pelleted cells were stored at − 20 °C.

To purify the expressed proteins, the cell suspension was subjected to sonication for 10 min after thawing, and the lysate was centrifuged for 45 min at 29,100×*g* (JA-17 rotor, Beckman, 4 °C). The supernatant was filtered through a membrane (0.45 μm pore size), and the filtrate was mixed with 1 mL of Ni^2+^ Sepharose resin (GE Healthcare), which was pre-equilibrated with 50 mM Tris/HCl pH 8.0. The mixture was incubated in closed gravity-flow column for 60 min at 4 °C while rotating. After the liquid was let through the column, the resin was washed with six column volumes of three solutions consisting of buffer (50 mM Tris/HCl pH 8.0) and varying concentrations of imidazole: 0, 5 and 10 mM. Subsequently, the bound proteins were eluted by applying 500 mM imidazole in 50 mM Tris/HCl pH 8.0. The obtained yellow elute was then applied to a PG-10 desalting column which was pre-equilibrated with 50 mM Tris/HCl pH 8.0, and the protein was eluted with the same buffer.

### Protein analyses

From the fractions obtained during purification (cell-free extract, flow-through, wash, purified), 10 μL samples of 2 mg/mL protein were taken. After addition of SDS loading dye and incubation at 95 °C for 5 min, the samples were spun down at 13,000×*g* for 1 min, and then loaded onto an SDS-PAGE gel (GenScript, USA) and run according to the recommendations of the gel supplier. A protein ladder (PageRuler pre-stained, Thermo Fisher) was also loaded. The gels were run in a Mini-PROTEAN® Tetra Vertical Electrophoresis Cell (Bio-Rad), and current was applied using a PowerPac^TM^ HC High-Current Power Supply (Bio-Rad), set at 120 V. When the blue front of the loading dye reached the bottom of the gel, the gel was removed from the chamber, rinsed with water and stained with Coomassie InstantBlue^TM^ (Fig. S1). Absorption spectra from 200 to 700 nm were taken of each purified fusion protein and diluted in buffer in a quartz cuvette (V-330 Spectrophotometer, JASCO). Using the obtained values at 280 and 441 nm, the protein concentration (ɛ_441_ = 14.0 mM^−1^ cm^−1^) and FAD ratio could be calculated.

### Activity assays

Kinetic measurements were done by following the formation or depletion of NADPH at 340 nm. After mixing enzyme (≤ 0.1 μM) with substrate in buffer (50 mM Tris/HCl pH 8.0), 200 μM NADP or NADPH was added, briefly mixed in a cuvette, and then the reaction was followed (V-330 Spectrophotometer, JASCO). For ADH activity, cyclohexanol (10 mM final concentration) was used as substrate, and for CHMO activity thioanisole (0.25 mM final concentration). The slopes of the initial 20 s were used to calculate the activity rates. The obtained slope value is expressed in absorption change per minute (Abs/min). This value was then divided by the extinction coefficient of NADPH (ɛ_340_ = 6.22 mM^−1^ cm^−1^), in accordance with Lambert-Beer law, giving a value in millimolar per minute. By dividing this value by the protein concentration in the reaction, the *k*_obs_ values were obtained. All measurements were done in duplicates or triplicates.

### Blue native PAGE and zymography

Native PAGE with all purified enzyme fusions was performed as described (Wittig et al. 2006), with some modifications. The cathode buffer consisted of 50 mM tricine, 7.5 mM imidazole, 0.002% *w*/*v* Coomassie blue G-250 (pH 7.0), the anode buffer of 25 mM imidazole (pH 7.0), and the sample loading dye of 50% glycerol and 0.5% Ponceau S (*w*/*v*). Samples were prepared by mixing 5 μL of fusion enzyme with 5 μL of loading dye, and they were loaded onto the gel (4–15% Mini-PROTEAN® TGX™, Bio-Rad). The gel electrophoresis was started at 60 V (10 mA, 1 W) at 4 °C, the voltage was increased by 10 every 30 min, and after 2 h, the gel was removed. The gel was washed with water, and then incubated with a zymography reaction solution (2 mM phenazine methosulfate (PMS), 0.3 mM nitro-blue tetrazolium (NBT), 0.5 mM NADP^+^, 50 mM cyclohexanol, in 50 mM Tris/HCl pH 9.0) for 20 min at 25 °C. The reaction mixture was removed when clear purple spots appeared in the gel, and the gel was subsequently stained with 0.02% (*w*/*v*) Coomassie blue G-250 in fixing solution (methanol:acetic acid:dH_2_O 40:10:50), and finally destained with 8% acetic acid solution.

### Gel filtration

Purified enzyme fusion samples were applied to a gel filtration column (Superdex 200 10/300 GL, GE Healthcare), which was equilibrated with buffer (50 mM Tris/HCl, 150 mM NaCl, pH 7.5) and mounted onto an ÄKTA pure system (GE Healthcare), and the flow rate was set at 0.6 mL/min of the same buffer. Through detection at 280 and 450 nm, the elution of the fusion enzymes could be followed in time. A gel filtration standard (Bio-Rad) was applied in the same way, and based on the elution times, a calibration curve was made, which was used to calculate the molecular weight of the fusion enzyme complexes.

### Melting point determination and stability

To determine the melting temperature of each fusion and the individual enzymes, samples were analyzed using the ThermoFluor® and ThermoFAD method (Forneris et al. 2009). Samples of 20 μL, containing 10 μM of enzyme, were incubated in a real-time PCR machine with a temperature gradient (20–99 °C, + 0.5 °C/min) while measuring fluorescence every 0.5 °C step. For the ThermoFluor® method, the dye SYPRO® orange (Thermo Fisher) was added to the samples. The determined value of melting temperature is the average value of two measurements. Long-term stability was evaluated by incubating 10 μM of enzyme in potassium phosphate buffer (100 mM, pH 8.0) at 37 °C and measuring the activity rates at various time points. For activity rate determination of the alcohol dehydrogenase, 10 mM of cyclohexanol and 0.20 mM NADP^+^ were used, and for the cyclohexanone monooxygenase activity, 0.25 mM of thioanisole with 0.20 mM NADPH was used. All measurements were performed in duplicate.

### Conversions

Small-scale biotransformations were performed in 2 mL Eppendorf tubes, with 0.5 mL of reaction mixture. The mixture consisted of fusion enzyme (5–20 μM), cofactor (200 μM NADP^+^), CAL-A lipase (10 mg/mL) and buffer (50 mM Tris/HCl pH 8.0 or 0.5 M potassium phosphate buffer pH 8.0). A control without fusion enzyme was run in parallel. The tubes with the reactions were incubated (37 °C, 600 RPM, ThermoMixer C, Eppendorf). During the incubation, substrate (stock 1 M cyclohexanol in buffer) was fed into the tubes with a syringe pump at a rate of 5 μL/h (Syringe Pump NE-4000). In later experiments, 10 mg/mL CAL-A lipase (c-LEcta) was included in the reactions. The samples were extracted three times with equivalent volume of ethyl acetate, including 1.0 mM of acetophenone as external standard. The pooled extract (1.5 mL) was dried with magnesium sulfate and then analyzed using GC-MS (HP-5 column, injection temperature 250 °C, oven temperature 40–130 °C, 5 °C/min).

### Submission of sequence

The codon-optimized nucleotide sequence coding for ADHMi from *Mesotoga infera* was submitted to GenBank according to the instructions of the website of GenBank. The amino acid sequence was originally obtained from NCBI (GenBank accession CCU83614.1). To order the corresponding synthetic gene, the amino acid sequence was submitted to GenScript, which produced the codon-optimized gene for expression in *E. coli*.

## Results

### Expression and purification of fusion enzymes

After cloning of each fusion construct (Table 1), transformed cells were grown for expression of the fusion enzymes. Expression was induced simultaneously with inoculation of the main culture, and this culture was incubated for 40 h at 24 °C. Purification of the yellow fusion enzymes was achieved through nickel affinity chromatography (Fig. S1). The imidazole was removed by applying the yellow fraction to a desalting column and subsequent elution with buffer. The purification yielded substantial protein amounts, which varied from 120 to 320 mg per liter culture. Similar amounts were obtained from expression and purification of the single ADH enzymes and TmCHMO, which suggests that the expression of the fusions is at least just as good (no data shown). In practice, expression with 50 mL cultures yielded a sufficient amount for biochemical experiments.

The degree of FAD cofactor in the fusion enzymes can be verified by measuring the absorption at 441 and 280 nm. Incorporation of this cofactor could be affected by the level of expression, as there could be insufficient FAD in the cell, and by hindrance from the fusion partner. Bound FAD is a sign of correctly folded flavoprotein. FAD ratios (A_280_/A_441_) of the fusions were found to range from 14 to 17, which indicates that the majority of each fused TmCHMO has FAD bound after purification (Table S2). From here on, the fusions will be abbreviated as follows: A-Tm (ADHA-TmCHMO), Mi-Tm (ADHMi-TmCHMO) and Tb-Tm (TbADH-TmCHMO) for the N-terminal ADH fusions (ADH-CHMO), and the fusions with a C-terminal ADH (CHMO-ADH) inversely (Tm-A, Tm-Mi and Tm-Tb).

### Activity screening

To determine the activity of each fusion enzyme, change in absorption at 340 nm was followed, as described in the “Materials and methods” section. The ADH oxidation rate was determined with 10 mM cyclohexanol as substrate. Cyclohexanone is also a substrate for the ADHs, for the reduction reaction with NADPH. Therefore, the BVMO activity of the fusion enzymes was measured using thioanisole (0.25 mM) as substrate (Table 2). The activity of TmCHMO on thioanisole is very similar to its activity on cyclohexanone (Romero et al. 2016).

**Table 2:** Fusion activity screening

Enzyme	ADH oxidation *k*_obs_ (s^−1^)	BVMO oxidation *k*_obs_ (s^−1^)
TmCHMO	–	1.05
ADHA	0.013	–
A-Tm	< 0.01	1.4
Tm-A	< 0.01	1.8
ADHMi	0.11	–
Mi-Tm	< 0.01	0.5
Tm-Mi	0.26	1.1
TbADH	1.35	–
Tb-Tm	1.08	1.9
Tm-Tb	0.28	1.6

Change in absorption at 340 nm measured at 25 °C in 50 mM Tris/HCl pH 8.0. Final substrate concentrations used: 10 mM cyclohexanol and 0.25 mM thioanisole. Cofactor concentration: 100 μM NADP^+^ or NADPH, respectively. For ADHMi and its fusions, 100 μM NAD^+^ was used for the alcohol oxidation. Reaction rates were calculated with protein concentrations determined from absorbance at 441 nm, using the extinction coefficient of TmCHMO (ɛ_441_ = 14.0 mM^−1^ cm^−1^)

It was gratifying to note that every fusion retained activity of the TmCHMO fusion partner. Overall, the BVMO activity was fairly consistent, aside from a two-fold decrease with Mi-Tm, and some fusions having slightly higher rates. Conversely, the alcohol oxidation activities of the fusions varied more severely. Neither A-Tm nor Tm-A showed any cyclohexanol oxidation activity. Interestingly, for the fusion Mi-Tm, no activity was observed, whereas Tm-Mi did show clear oxidation rates. With the fusions of TbADH, the reverse was observed: Tb-Tm has a wild-type level of activity, whereas Tm-Tb had a four-fold lower activity.

Previous studies on the cascade reaction from cyclohexanol to ɛ-caprolactone (Fig. 1) have used AcCHMO and pointed out that it is inactivated and inhibited by cyclohexanol, and by ɛ-caprolactone (Mallin et al. 2013; Staudt et al. 2013). It is important to know whether this also applies to TmCHMO, since inhibition could negatively affect the productivity of the cascade. Inhibition of TmCHMO was studied at several concentrations of inhibitor, and thioanisole was used as substrate to determine the activity. The single TmCHMO as well as the Tm-A fusion were tested. Regarding product inhibition, a 50% reduction of activity was observed in the presence of 66 mM of ε-caprolactone, and an 80% reduction at 133 mM. Results with cyclohexanol showed that presence of 2 mM was sufficient to cause a decrease in activity of 75% (data not shown). Like AcCHMO, TmCHMO also suffers from inhibition, though it is more robust.

### Oligomerization of fusion enzymes

Alcohol dehydrogenases typically form dimers or tetramers, which in most cases is needed for stability and/or activity. Two techniques were used to investigate the oligomerization behavior of the fusion enzymes: blue native PAGE (BN-PAGE) combined with zymography and gel filtration chromatography.

**Fig. 2:**
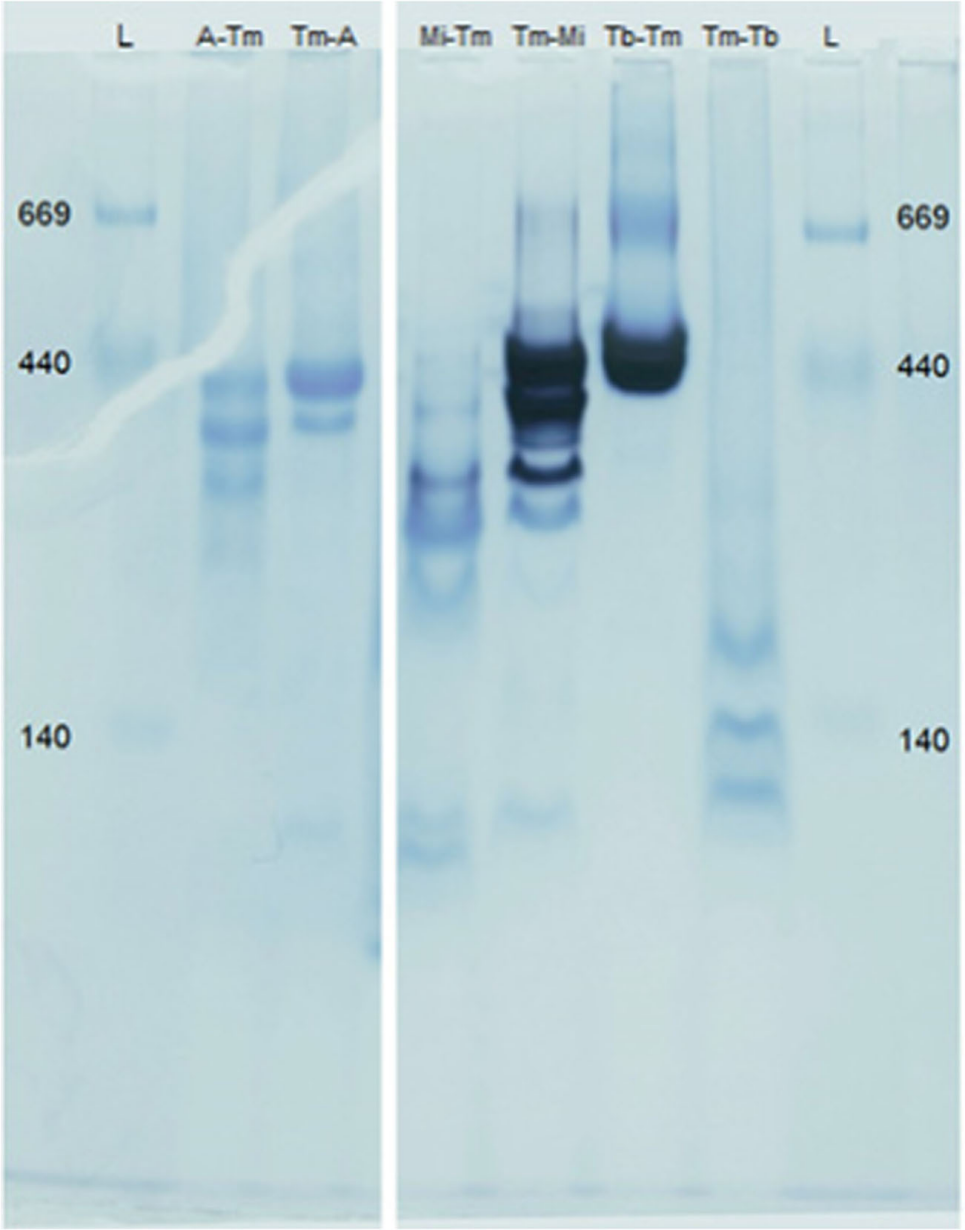
Blue native PAGE stained with Coomassie and zymography simultaneously. The HMW Native Marker ladder (GE Healthcare) values are given in kDa. The dark, purple stain results from the zymography, whereas the lighter, blue stain is caused by the Coomassie blue G-250 treatment

After BN-PAGE, the gel was subjected to zymography, which causes active ADHs to show a dark purple band (Fig. 2 and Fig. S2). The following enzymes showed no activity: both of the ADHA fusions and Tm-Tb. Minor activity was seen for Mi-Tm, whereas the other enzymes showed clear dark bands (Tm-Mi and Tb-Tm). Coomassie staining revealed that, aside from the two ADHA fusions, every inactive fusion also had a higher electrophoretic mobility compared to their active counter-parts, which suggests possible structural changes. When comparing two fusions consisting of identical proteins, e.g. Mi-Tm with Tm-Mi, it seems that each active fusion has lower electrophoretic mobility, indicative of a distinct structural organization.

Protein size was estimated using gel filtration chromatography (Fig. S3). In accordance with results from the BN-PAGE, oligomers above 400 kDa were determined for the fusions A-Tm, Tm-A, Tm-Mi and Tb-Tm, which supports the impression that these fusion enzymes form tetramers. Each individual ADH had an elution time corresponding to homodimers. The fusion enzyme Tm-Tb has a main peak at its monomer size, while for the Mi-Tm fusion, a trimer size of about 280 kDa was observed.

### Enzyme stability

Since the fusion enzymes consist of a single polypeptide chain, the stability and the tendency to unfold could be changed compared to the individual enzymes. For each fusion enzyme, the melting point was determined by using ThermoFluor and ThermoFAD (Table S2). With the latter, the release of the FAD from TmCHMO upon unfolding could be detected, while with ThermoFluor, any protein unfolding gives a signal by binding of a dye (SYPRO orange). The more stable ADHs transfer some stability to TmCHMO (Δ*T*_m_ of up to +3 °C). The results from ThermoFluor have values similar to those from ThermoFAD. This is due to the lower melting point of TmCHMO compared to the ADHs.

The fusions Mi-Tm, Tm-Mi and Tb-Tm display a second melting peak in the ThermoFluor assay (Fig. S4). In each case, the second peak is some degrees lower than the single ADH melting point. For instance, the *T*_m_ of the single ADHMi is 69 °C, while the second peak of the Tm-Mi fusion is at 65 °C. This suggests that in those fusions each fusion partner unfolds more independently than the individual ADH.

To evaluate long-term stability, Tb-Tm was incubated at 37 °C for 42 h (Fig. S6). Residual ADH and TmCHMO activity was determined by monitoring the change in absorption at 340 nm after addition of substrate and nicotinamide cofactor. The long-term incubation was also carried out for TbADH and TmCHMO as separate enzymes. The fusion retained slightly more activity: after 42 h, the fusion had 92% of the ADH and BVMO activity, whereas the separate TbADH and TmCHMO retained 88 and 82% activity, respectively.

### Conversions

The most active and stable fusion was selected to perform small-scale conversions with cyclohexanol. Based on the results from the other experiments, the Tb-Tm fusion was selected. Biocatalytic production of ε-caprolactone was performed by combining the Tb-Tm fusion with NADP^+^ and cyclohexanol (Table 3). The total volume of each conversion was 0.5 mL. After conversion for 24 h at 37 °C, the reaction sample was extracted three times with ethyl acetate, dried with magnesium sulfate and then analyzed using GC-MS (HP-5 column) (Fig. S5). The percentage of conversion is defined by depletion of substrate (cyclohexanol) and intermediate (cyclohexanone), compared to the control with enzyme.

**Table 3:** Conversions

Enzyme(s)	Substrate (mM)	Time (h)	Temperature (°C)	Conversion	TON
Tb-Tm (15 μM)	200	24	37	> 99%	13,333
TbADH (15 μM) + TmCHMO (15 μM)	200	24	37	42%	5600
Tb-Tm (5 μM) + TbADH (5 μM)	200	24	37	95.8%	19,160
Tb-Tm (5 μM) + TmCHMO (5 μM)	200	24	37	> 99%	20,000
Tb-Tm (20 μM)	500	48	30	41.5%	10,375
TbADH (20 μM) + TmCHMO (20 μM)	500	48	30	33%	8250

Conversions included substrate feeding (cyclohexanol, 1 M) at 5 μL/h, and 10 mg/mL CAL-A lipase, in 0.5 M potassium phosphate buffer pH 8.0. Conversion is calculated based on the depletion of cyclohexanol (and intermediate, cyclohexanone), from the GC-MS measurements. TON = Turnover number, per fusion molecule, regarding the transformation of cyclohexanol to ε-caprolactone as one turnover

Initial experiments (15 μM Tb-Tm, 200 μM NADP^+^, 24 h, 37 °C) on low (10 mM) substrate concentration gave 100% conversion, while high (> 100 mM) substrate loadings gave near to 0% conversion. The latter result can be explained by the TmCHMO inhibition by cyclohexanol. Therefore, substrate (1 M concentration) was fed into the reaction with a syringe pump at 5 μL/h (Syringe Pump NE-4000) to attain more conversion with higher substrate loading. By this, a conversion of 64% could be achieved in 24 h. To investigate whether product inhibition was the main cause of the incomplete conversion, CAL-A lipase was included in the reactions, as described in a previous study (Schmidt et al. 2015). This lipase can convert ε-caprolactone to oligo-caprolactone, thereby reducing product inhibition. After incubation of the reaction including CAL-A (10 mg/mL) for 24 h, the conversion reached 80.5%. The pH of the reaction drastically decreased during the conversion; from pH 8.0 to 5.0 after 24 h, which is likely due to hydrolysis by the CAL-A lipase. To maintain pH, experiments were continued in a high concentration of potassium phosphate buffer (0.5 M, pH 8.0), which was tested with each enzyme for activity and stability (data not shown). This enabled us to get > 99% conversion of 200 mM cyclohexanol within 24 h. If a turnover is defined as the transformation of one molecule of cyclohexanol to one molecule of ɛ-caprolactone, then each fusion molecule performed 13,333 turnovers (TON), and NADP^+^ 1000 turnovers. Intriguingly, a parallel reaction was performed with the TbADH and TmCHMO as separate enzymes, which gave a conversion of only 42%.

To investigate which of the two enzymes is rate-limiting, conversions with the fusion (5 μM) and an additional amount of either the TbADH or TmCHMO (5 μM) were made, such that the ratio between the two enzymes was 2:1 or 1:2 (Table 3). The reaction with additional TbADH reached 95.8% conversion after 24 h, whereas the one with TmCHMO reached > 99%, showing that the TmCHMO is the slowest fusion partner.

## Discussion

In this study, we designed ADH–TmCHMO fusion constructs, expressed the genes in *E. coli* cells and then purified the fusion enzymes. Good amounts of FAD-containing fusion enzymes could be produced and purified. For studies on multi-enzyme reactions, the required enzymes are typically produced individually. By enzyme fusion, a single expression and purification yields bifunctional enzymes, thereby greatly reducing the effort and possibly improving the expression. Moreover, in some cases, a fusion can perform successive reactions more efficiently, as found in various studies so far (Iturrate et al. 2010; Jeon et al. 2015; Lerchner et al. 2016; Peters et al. 2017).

However, we have found that this co-expression does not always produce fully active fusion enzymes. In particular, some of the fusions lacked ADH activity, while the TmCHMO retained activity in each of the six fusions. One of the ADHs that was fused is the ADHA from *P. furiosus*, which has extraordinary thermostability with a reported half-life of 22.5 h at 90 °C (Van der Oost et al. 2001). Yet, its catalytic rates are relatively poor, in particular at lower temperatures. Considering this fact, it was not surprising to find that ADHA and its fusions were unable to oxidize cyclohexanol at 25 °C. Unlike this lack of activity of ADHA and its fusions, the reason for the inactivity of Mi-Tm is more obscure. The individual ADHMi enzyme, and its fusion Tm-Mi, are capable of oxidizing cyclohexanol. Therefore, the lack of activity is probably caused by the orientation, i.e. the fusion of the BVMO at the C-terminal side of the ADH. Incidentally, ADHMi is a short-chain dehydrogenase (SDR superfamily, with 250 amino acids on average). A recent study pointed out that for *Lactobacillus kefir* RADH, also an SDR, a C-terminal His-tag was detrimental for its activity (Peters et al. 2017). Furthermore, levodione reductase from *Leifsonia aquatica* had 40–70% decrease in activity with a C-terminally fused aminotransferase, depending on the linker length (Lerchner et al. 2016). The findings point to a common trend for SDR enzymes, where a fusion tag or enzyme at the C-terminal side of the SDR is disruptive for its activity.

In addition to lack of activity, the fusion Mi-Tm showed a smaller oligomeric size based on gel filtration, indicating that it forms a trimer. A plausible explanation for this aberration is that the region at the C-terminal side of the ADHMi is part of the oligomerization interface. The association between four ADH subunits would be hindered by the BVMO domains that are located at this interface. Consequently, the ADH is unable to form a particular oligomer, which could be needed for stability and activity (Breiter et al. 1994). On the other hand, the Tm-Mi fusion shows a tetramer size with the gel permeation experiments, and activity with zymography and spectrophotometric assay.

For the fusions with TbADH, a medium-chain reductase (MDR) enzyme, the situation is reversed. In this case, the fusion Tb-Tm has wild-type activity, while Tm-Tb has a four-fold lower ADH activity. The effect is less drastic compared to that of the ADHMi. The finding is in line with a study on phosphite dehydrogenase (PTDH)-BVMO fusions (Torres Pazmiño et al. 2008). In that study, the PAMO-PTDH fusion has 1.5-fold lower PTDH activity compared to the PTDH-PAMO fusion. Possibly, MDR enzymes favor the latter orientation in general, while showing a decrease in activity in the reverse orientation. As with Mi-Tm, the low activity of Tm-Tb correlates with a different oligomeric state, indicating a monomer, while the more active Tb-Tm seems to form a tetramer.

Previous studies on biocatalytic ε-caprolactone production have used AcCHMO, which was found to be inhibited by cyclohexanol and by ε-caprolactone. We found that, compared to AcCHMO, the TmCHMO, though more thermostable, is also prone to inhibition. This property is unfortunate for the cascade reaction with Tb-Tm, as the *K*_M_ for cyclohexanol of the TbADH (7 mM) is higher than the estimated *K*_I_ for cyclohexanol (< 2 mM) of TmCHMO. Based on the conversions in which either enzyme was added in a 2:1 ratio, the BVMO was shown to be the bottleneck of the cascade reaction. Therefore, the TmCHMO inhibition is an important aspect to investigate in future studies, in order to improve productivity of cascade reactions with these fusion enzymes.

The fusion Tb-Tm was selected to perform the cascade reaction to produce ε-caprolactone, as it showed the highest activity on cyclohexanol. Multiple adjustments had to be made in order to get more conversion. Inhibition of TmCHMO by substrate and product needed to be addressed, as well as the decrease in pH, which is probably the result of ε-caprolactone hydrolysis by CAL-A, as it would form 6-hydroxyhexanoic acid. The decrease in pH reduces the stability and the rates of the enzymes. In particular, the oxidation rate of ADHs is dependent on pH, as the deprotonation of the alcohol is favored at higher pH, which promotes oxidation of the alcohol. With substrate feeding, addition of CAL-A lipase, and by using a strong buffer, > 99% of 200 mM cyclohexanol could be converted in 24 h. This result shows that the fusion can continuously perform oxidations by recycling of the cofactor, with a turnover number (TON) of > 13,000 for the fusion enzyme. In a study with a similar cascade, in which AcCHMO was used, the enzyme reached a TON of approximately 5800 (Bornadel et al. 2016), which suggests that the superior stability of TmCHMO enables higher turnover numbers. The fusion seems to perform better than the combined individual enzymes. Several factors could play a role in this difference: slightly worse stability of the non-fused TmCHMO (Table S2, Fig. S6), higher activity of the TmCHMO in the Tb-Tm fusion (Table 2), and possibly a benefit of the co-localization of the two enzymes, as the cyclohexanone and NADPH formed by the ADH could directly migrate to the active site of the TmCHMO.

Conversions with relatively high final substrate concentrations showed difficulties due to solubility, enzyme stability and inhibition. When the activity of the TmCHMO decreases, while the substrate feeding rate remains equal, the process will eventually stop due to complete inhibition of TmCHMO by the accumulated cyclohexanol. This effect also explains why factors, such as small differences in stability and activity, combined could lead to incomplete conversions, as was observed with the separate enzymes. It might be possible to convert more substrate if the substrate feeding flow is decreased in proportion to the decrease of active enzyme, or if additional enzyme is added. The intricate balancing of the kinetic parameters and stability of the three enzymes involved in the cyclohexanol to 6-hydroxyhexanoic acid cascade reaction was recently investigated (Scherkus et al. 2017). The authors used computational simulations, as well as experiments, to find the most favorable approach and conditions for large-scale conversions. In a similar cascade, involving an enoate reductase in addition to ADH and CHMO, kinetic modeling was applied to identify bottlenecks for in vivo biotransformations (Milker et al. 2017). The kinetic models described could be applied to the robust Tb-Tm fusion presented herein, to further optimize the cascade towards an industrial process for ɛ-caprolactone production. Notably, as both the ADH and CHMO can accept a broad range of substrates, the fusions can be used for the production of various interesting esters or lactones through analogous cascade reactions.

## Electronic supplementary material

ESM 1 (PDF 353 kb)
