# Supplementary material for: Coupled reactions by coupled enzymes: alcohol to lactone cascade with alcohol dehydrogenase–cyclohexanone monooxygenase fusions
Source: Appl Microbiol Biotechnol. 2017 Sep 15;101(20):7557–65. doi: 10.1007/s00253-017-8501-4 (PMC5624969; doi:10.1007/s00253-017-8501-4)
Supplement: Supplementary file 1 — (PDF 353 kb) [file 253_2017_8501_MOESM1_ESM.pdf]

“Coupled reactions by coupled enzymes: Alcohol to Lactone cascade with Alcohol Dehydrogenase – Cyclohexanone Monooxygenase Fusions” – Supplementary material

Applied Microbiology and Biotechnology

Friso S. Aalbers and M.W. Fraaije

Laboratory of Biochemistry, Groningen Biomolecular Sciences and Biotechnology Institute, University of Groningen, Nijenborgh 4, 9747 AG Groningen, The Netherlands

E-mail: m.w.fraaije@rug.nl

Table S1. Oligonucleotide primers for fusion cloning. Nucleotide order is from 5’ to 3’. Fusions were made with either an *adh* as first gene (‘begin’), with the TmCHMO gene downstream, or as second gene (‘end’), after the *TmCHMO* gene.

begin_AdhA_NdeI_F	GGAATTCCATATGAAGGTTGCCGTAATTACTGGGG
begin_AdhA_XhoI_R	CTGACTCGAGTACTCAGGTTTTTGATAAATTGAGCG
end_AdhA_PvuII_F	GCTTCAGCTGGTATGAAGGTTGCCGTAATTACTGGGG
end_AdhA_ApaI_R	GACAGGGCCCTTAATACTCAGGTTTTTGATAAATTGAGCG
begin_AdhMi_NdeI_F	GGAATTCCATATGAAGCGTTTTGAGGGCAAGGTGG
begin_AdhMi_XhoI_R	CTGACTCGAGCCCTTCGCGGTATAACCACCATC
end_AdhMi_HindIII_R	CTCAAGCTTACTTCGCGGTATAACCACC
end_AdhMi_PvuII_F	GGAATTCCAGCTGGTATGAAGCGTTTTGAGGGCAAG
begin_AdhT_NdeI_F	GGAATTCCATATGAAAGGTTTCGCC
begin_AdhT_XhoI_R	CTGACTCGAGGCCAGAATAACAACTGG
end_AdhT_PvuII_F	GCTTCAGCTGGTATGAAAGGTTTCGCCATG
end_AdhT_HindIII_R	CTCAAGCTTACGCCAGAATAACAACTG
begin_TmCHMO_NdeI_F	GGAATTCCATATGAGCACCACCCAGACCC
begin_TmCHMO_XhoI_R	CTGACTCGAGGCCACCGCTTGCGCACGTTCACC
end_TmCHMO_PvuII_F	GCTTCAGCTGGTATGAGCACCACCCAGACCC
end_TmCHMO_HindIII_R	CTCAAGCTTACGCCACCGCTTGCGCACGTTCA

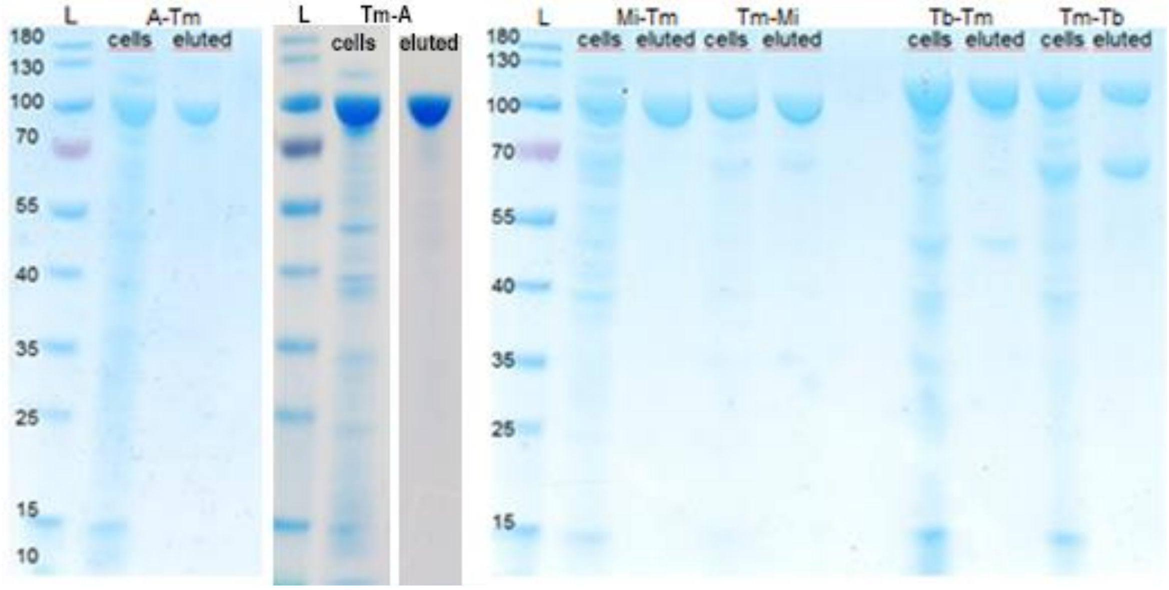

Figure S1. SDS-PAGE of the purification of 6 fusion constructs. Values of PageRuler sizes expressed in kDa. The ‘eluted’ fraction is the protein sample obtained after the elution with 500 mM imidazole.

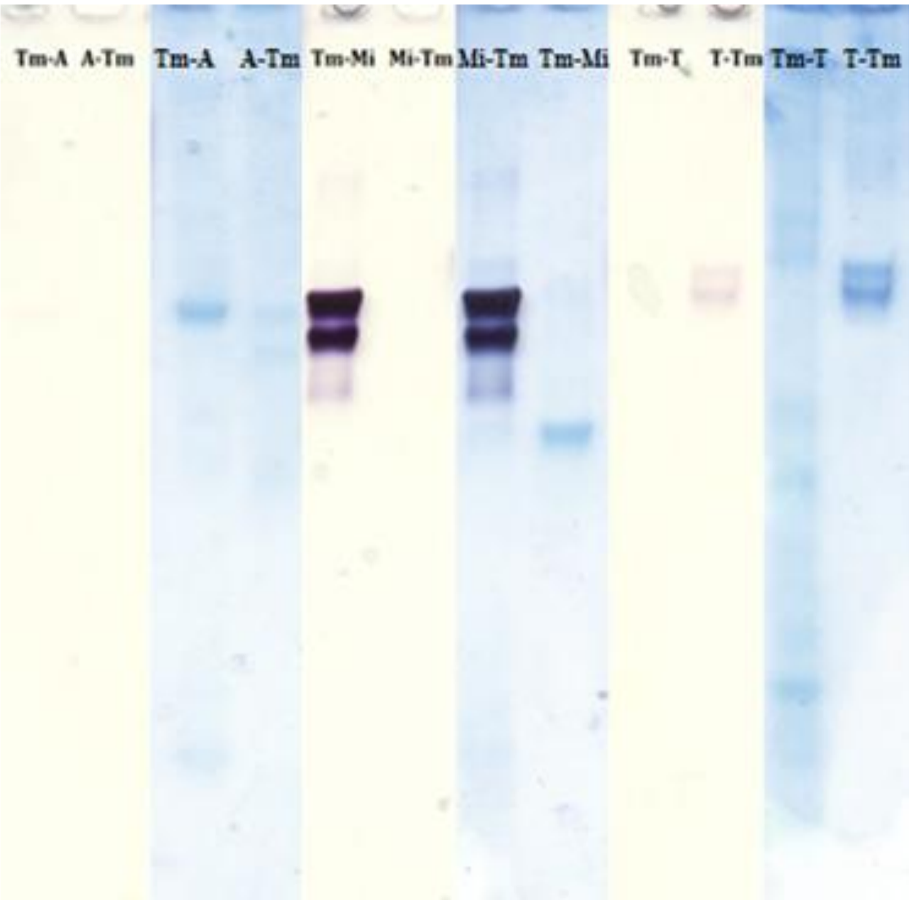

Fig. S2 Blue native-PAGE, first stained with zymography (left tile of every fusion), and afterwards stained with Coomassie blue (right tile).

Fig. S3 Gel filtration chromatograms of the fusion enzymes, with elution volumes on the X-axis (mL) and the relative absorption at 280 nm on the Y-axis. Elution volumes lower than 11 mL correspond to a kDa ≥ 400, and volumes more than 13 mL correspond to molecular weights ≤ 100 kDa, based on calibration curve made from standards (BioRad). A: A-Tm (grey) and Tm-A (black). B: Mi-Tm (grey) and Tm-Mi (black). C: Tb-Tm (grey) and Tm-Tb (black).

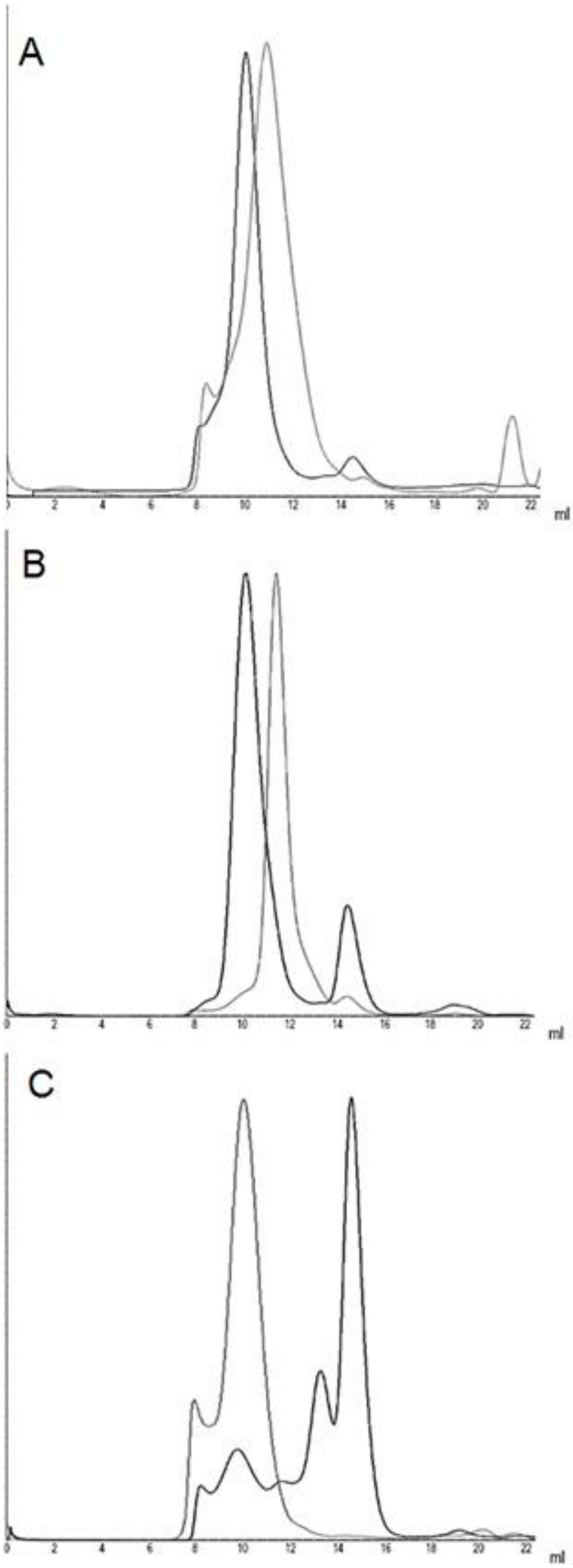

Table S2. Fusion enzyme data

Enzyme	FAD ratio	Expression (mg/L culture)	Melting point [a] (°C)	Melting point [b] (°C)	Melting point [c] (°C)
ADHA	-	-	-	98.5	-
A-Tm	17.4	120	48.0	48.5	-
Tm-A	-	-	48.5	53.0	-
ADHMi	-	-	-	69.0	-
Mi-Tm	15.9	180	49.5	50.0	65.5
Tm-Mi	15.0	290	48.8	50.3	65.0
TbADH	-	-	-	93.0	-
Tb-Tm	16.2	320	48.8	51.5	85.3
Tm-Tb	14.4	320	49.0	49.5	-

FAD ratio (A_{280}/A_{441}), expression, ThermoFluor® and ThermoFAD results. Melting point [a] is the ThermoFAD result, [b] is the ThermoFluor® value of the first peak, [c] is the Thermofluor second peak.

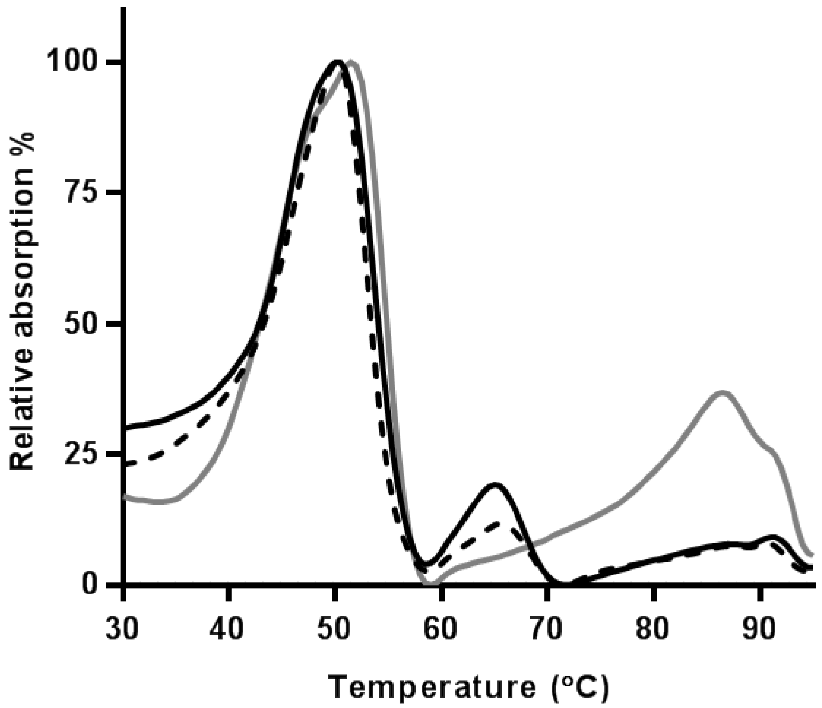

Fig. S4 ThermoFluor® of the samples showing second peaks. Black line = Mi-Tm, dashed black line = Tm-Mi, grey line = Tb-Tm. The graphs represent the average of duplicate experiments.

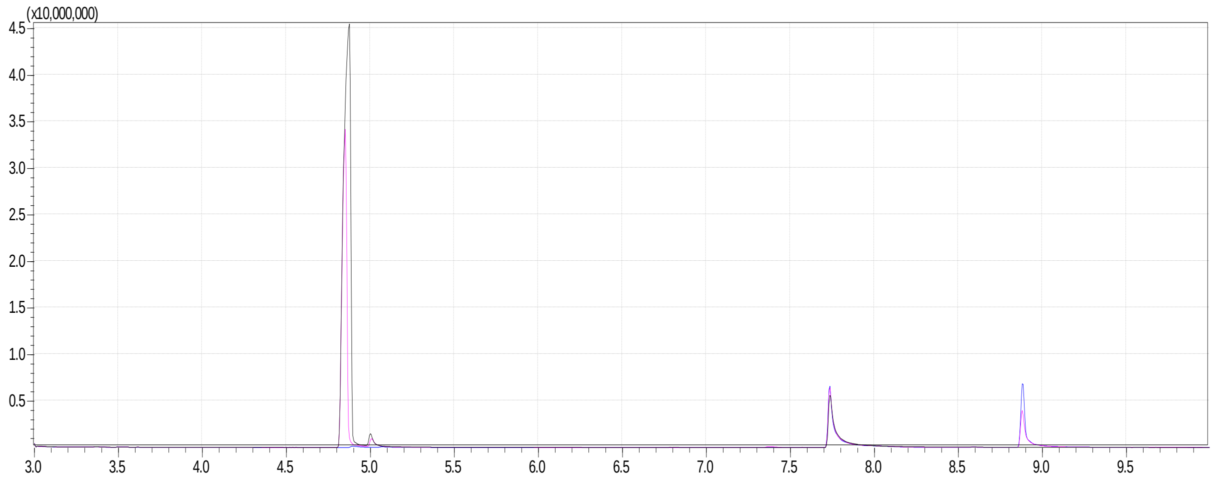

Fig. S5 GC-MS result of the conversion of cyclohexanol to ε-caprolactone after 24 hours (Table 3, first row). Black = control, pink = TbADH with TmCHMO separately, blue = TbADH-TmCHMO fusion. The first peak, with a retention time of 4.8 minutes, corresponds to cyclohexanol. The second peak (5.0) is cyclohexanone. The external standard (acetophenone) has a peak at 7.7 min. The last peak, at 8.9 minutes, corresponds to ε-caprolactone.

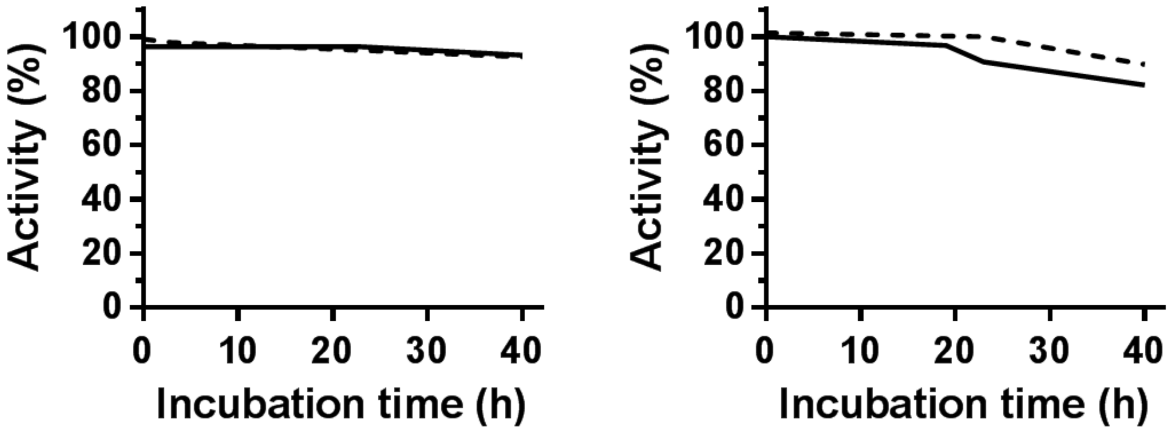

Fig. S6 Stability of ADH activity (left) and CHMO activity (right) over time at 37 °C, 0.5 M KPi pH 8.0. The dashed lines represent the activity of the Tb-Tm fusion, the bold line the activity of the separate TbADH (left) or TmCHMO (right).
